# Hepatobiliary-Specific MRI Contrast Agent Detection of Subvesical Duct (Luschka’s) Injury Post-Laparoscopic Cholecystectomy: A Case Report

**DOI:** 10.5334/jbsr.3532

**Published:** 2024-02-20

**Authors:** Ahmet Bozer

**Affiliations:** 1 Department of Radiology, Bozyaka Educatıon and Research Hospıtal, İzmir, Turkey

**Keywords:** Bile duct injury, Gadolinium DTPA, contrast agent, Luschka’s duct, MRI

## Abstract

Post-laparoscopic cholecystectomy bile duct injuries, especially involving Luschka’s duct, are concerning. Biliary tree anomalies and the efficacy of intravenous administration of Gd-EOB-DTPA-enhanced magnetic resonance imaging (MRI) in detecting bile leakage are reported based on a case.

*Teaching Point:* Hepatobiliary-specific MRI-contrast agents prove valuable for noninvasive assessment of bile leakage after cholecystectomy.

## Introduction

Bile duct injuries are among the most frequent and significant complications of laparoscopic cholecystectomy. The reported incidence ranges between 0.4% and 0.8%, while for traditional cholecystectomy ranges from 0.1% to 0.3% are reported [1]. The most prevalent injury involves the cystic duct, followed by injuries to neighboring structures such as Luschka’s duct [2].

Luschka ducts are clinically significant anatomical variants of the biliary tree due to potential surgical injury risks with cholecystectomy, hepatic resections, and interventional procedures [3]. Despite a reported 4% prevalence, there might be an underestimation due to a lack of routine imaging-based assessment.

## Case Presentation

**Figure 1:**
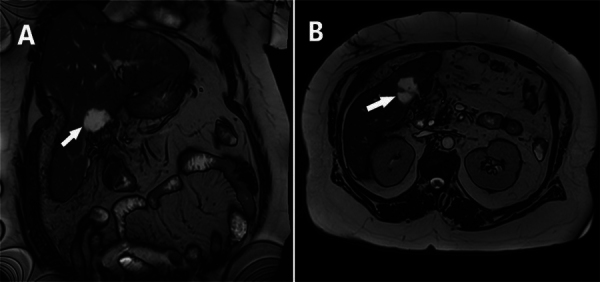
Coronal (A) and axial (B) T2-weighted MRI images demonstrate a loculated collection (arrow) in the gallbladder bed.

**Figure 2:**
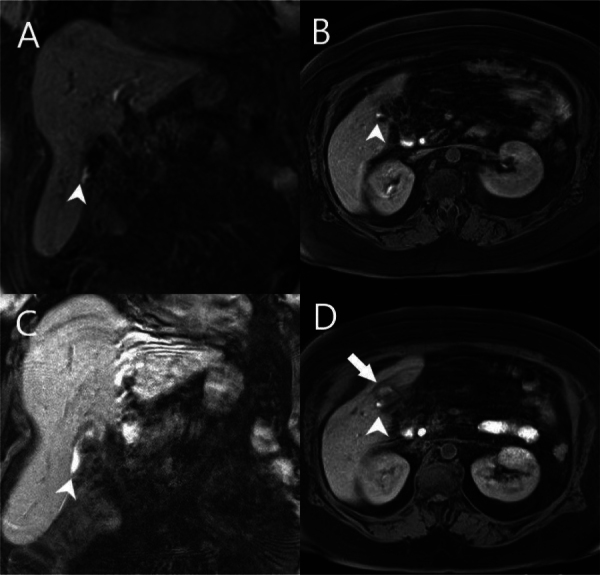
Gd-EOB-DTPA-enhanced T1-weighted 3D GRE images at 50 minutes (A: coronal, B: axial) and 2 hours (C: coronal, D: axial) post-injection. The subvesical bile duct (arrowhead) supplies the biloma with hepatobiliary contrast-enhanced bile (arrow).

**Figure 3:**
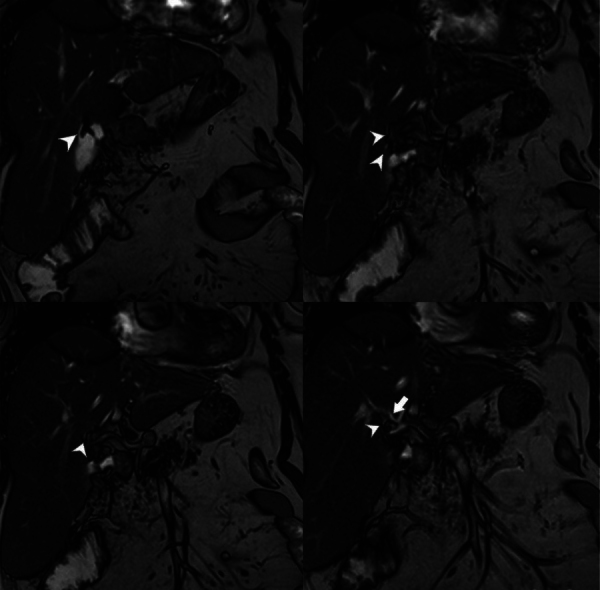
Consecutive series of T2-weighted coronal MRI images illustrating the course of the subvesical bile duct (arrowhead) and its merging with the right hepatic duct (arrow).

A 57-year-old woman was referred to the emergency department with abdominal pain, nausea, and vomiting 4 days post-laparoscopic cholecystectomy. Clinical examination revealed tenderness in the right upper quadrant. Serum inflammatory markers were elevated, while functional liver and pancreatic tests were normal. Abdominal ultrasound identified a 2 cm fluid collection in the gallbladder bed, confirmed at computed tomography (CT). With a preliminary diagnosis of biloma, a magnetic resonance imaging (MRI) with a hepatobiliary-specific (HBS) contrast agent, gadoxetate disodium (Gd-EOB-DTPA; Primovist—Eovist; Bayer Healthcare, Germany) was performed to detect the location of the leakage. On contrast-enhanced MRI, the accessory Luschka’s bile duct leaking into the biloma was visible 50 minutes later, more clearly after 2 hours, and illustrating the connection with the right hepatic duct (Figures 1–3).

Under ultrasound guidance, 15 cc of dense green fluid was aspirated, and a 10 F drainage catheter was inserted. Endoscopic retrograde cholangiopancreatography (ERCP) showed no cystic duct leakage. A sphincterotomy was performed, and a 10 Fr/10 cm biliary stent was inserted.

After 6 days, the drain was removed, and the patient was discharged symptom free on day 15.

## Discussion

Bile duct injuries are classified according to the Bismuth or Strasberg systems. The Bismuth classification is based on the location of biliary strictures relative to the confluence, but it doesn’t cover the entire spectrum of injuries. Strasberg et al. [4] expanded the Bismuth classification to include additional types of extrahepatic bile duct injuries (Table 1) [5]. Type A is the most common injury, often occurring after laparoscopic cholecystectomy and involving leakage from the cystic duct or Luschka bile ducts, as in our case (Figure 2).

**Table 1:** Strasberg classification of bile duct injuries

Type	Criteria
A	Leaks from the cystic duct or the bile ducts of Luschka
B	Occlusion of aberrant right hepatic ducts
C	Transection without ligation of aberrant right hepatic ducts
D	Lateral injuries to major bile ducts
E	Subdivided per the Bismuth classification into E1–E5

Luschka ducts are accessory ducts along the gallbladder fossa, typically connecting to the right hepatic duct (Figure 3) [4]. The terminology for this anatomical variant includes accessory biliary ducts, vasa aberrantia, subvesical, or subvesicular. To address the variability in structures in the gallbladder fossa and the lack of standardized terminology, Schnelldorfer et al. [3] introduced the “subvesicular bile duct” classification based on a systematic review. This includes both Luschka and hepaticocholecystic ducts into four types: Type 1—superficial variants of sectorial and segmental bile ducts, Type 2—superficial or intercommunicating ducts (the true Luschka duct), Type 3—hepaticocholecystic ducts, and Type 4—aberrant bile ducts [3].

MRI and magnetic resonance cholangiopancreatography (MRCP) provide a noninvasive alternative to ERCP for detecting bile leakage. Fat-saturated T1-weighted MR-contrast sequences with HBS agents such as gadobenate dimeglumine (Gd-BOPTA) (MultiHance, Bracco Diagnostics, Italy) or Gd-EOB-DTPA enable the functional evaluation of the biliary system and visualization of patent leaks after 15 to 90 minutes [6]. Delayed imaging after 90 to 120 minutes after Gd-BOPTA administration is required, while a shorter interval of 10 to 20 minutes is sufficient for Gd-EOB-DTPA [6]. Imaging with a delay of at least 90 minutes is likely to be more effective than the conventional 20-minute delay in confirming bile leakage [7].

In this case, at 50 minutes post-injection, the subvesical bile duct feeding the biloma was detected, more clearly observable around 2 hours after injection.

Contrast-enhanced MRCP is recommended by the 2020 guidelines of the World Society of Emergency Surgery (WSES) for bile leak detection, based on its superior performance compared to conventional MRCP [8].

## Conclusion

HBS-MR contrast agents are crucial for detecting cholecystectomy-related bile leakage. Delayed imaging up to approximately 2 hours may be necessary for an accurate diagnosis.

## References

[r1] Mangieri CW, Hendren BP, Strode MA, Bandera BC, Faler BJ. Bile duct injuries (BDI) in the advanced laparoscopic cholecystectomy era. Surg Endosc. 2019;33(3):724–730. DOI: 10.1007/s00464-018-6333-7. 30006843

[r2] Pioche M, Ponchon T. Management of bile duct leaks. J Visc Surg. 2013;150(3 Suppl):S33–8. DOI: 10.1016/j.jviscsurg.2013.05.004. 23791984

[r3] Schnelldorfer T, Sarr MG, Adams DB. What is the duct of Luschka?—A systematic review. J Gastrointest Surg. 2012;16(3):656–662. DOI: 10.1007/s11605-011-1802-5. 22215244

[r4] Strasberg SM, Hertl M, Soper NJ. An analysis of the problem of biliary injury during laparoscopic cholecystectomy. J Am Coll Surg. 1995;180(1):101–125. 8000648

[r5] Lau WY, Lai EC. Classification of iatrogenic bile duct injury. Hepatobiliary Pancreat Dis Int. 2007;6(5):459–463. 17897905

[r6] Seale MK, Catalano OA, Saini S, Hahn PF, Sahani D V. Hepatobiliary-specific MR contrast agents: Role in imaging the liver and biliary tree. Radiographics. 2009;29(6):1725–1748. DOI: 10.1148/rg.296095515. 19959518

[r7] Cieszanowski A, Stadnik A, Lezak A, et al. Detection of active bile leak with Gd-EOB-DTPA enhanced MR cholangiography: Comparison of 20–25 min delayed and 60–180 min delayed images. Eur J Radiol. 2013;82(12):2176–2182. DOI: 10.1016/j.ejrad.2013.08.021. 24012454

[r8] de’Angelis N, Catena F, Memeo R, et al. 2020 WSES guidelines for the detection and management of bile duct injury during cholecystectomy. World J Emerg Surg. 2021;16(1):1–27. DOI: 10.1186/s13017-021-00369-w. 34112197 PMC8190978

